# Empagliflozin suppresses urinary mitochondrial DNA copy numbers and interleukin-1β in type 2 diabetes patients

**DOI:** 10.1038/s41598-022-22083-6

**Published:** 2022-11-09

**Authors:** Haekyung Lee, Hyoungnae Kim, Jin Seok Jeon, Hyunjin Noh, Rojin Park, Dong Won Byun, Hye Jeong Kim, Kyoil Suh, Hyeong Kyu Park, Soon Hyo Kwon

**Affiliations:** 1 Division of Nephrology, Department of Internal Medicine, Soonchunhyang University Seoul Hospital, 59 Daesagwan-ro, Yongsan-gu, Seoul, 04401 Republic of Korea; 2 Hyonam Kidney Laboratory, Soonchunhyang University Seoul Hospital, 59 Daesagwan-ro, Yongsan-gu, Seoul, 04401 Republic of Korea; 3 Department of Laboratory Medicine, Soonchunhyang University Seoul Hospital, 59 Daesagwan-ro, Yongsan-gu, Seoul, 04401 Republic of Korea; 4 Division of Endocrinology and Metabolism, Department of Internal Medicine, Soonchunhyang University Seoul Hospital, 59 Daesagwan-ro, Yongsan-gu, Seoul, 04401 Republic of Korea

**Keywords:** Endocrine system and metabolic diseases, Biomarkers

## Abstract

Sodium-glucose co-transporter 2 (SGLT2) inhibitors improve cardiovascular and renal outcomes in type 2 diabetes mellitus (T2DM) patients. However, the mechanisms by which SGLT2 inhibitors improve the clinical outcomes remain elusive. We evaluated whether empagliflozin, an SGLT2 inhibitor, ameliorates mitochondrial dysfunction and inflammatory milieu of the kidneys in T2DM patients. We prospectively measured copy numbers of urinary and serum mitochondrial DNA (mtDNA) nicotinamide adenine dinucleotide dehydrogenase subunit-1 (mtND-1) and cytochrome-c oxidase 3 (mtCOX-3) and urinary interleukin-1β (IL-1β) in healthy volunteers (*n* = 22), in SGLT2 inhibitor-naïve T2DM patients (*n* = 21) at baseline, and in T2DM patients after 3 months of treatment with empagliflozin (10 mg, *n* = 17 or 25 mg, *n* = 4). Both urinary mtDNA copy numbers and IL-1β levels were higher in the T2DM group than in healthy volunteers. Baseline copy numbers of serum mtCOX-3 in the T2DM group were lower than those in healthy volunteers. Empagliflozin induced marked reduction in both urinary and serum mtND-1 and mtCOX-3 copy numbers, as well as in urinary IL-1β. Empagliflozin could attenuate mitochondrial damage and inhibit inflammatory response in T2DM patients. This would explain the beneficial effects of SGLT2 inhibitors on cardiovascular and renal outcomes.

## Introduction

Diabetes mellitus (DM) is the leading cause of chronic kidney disease and end-stage kidney disease, as well as a well-recognized risk factor for cardiovascular diseases and mortality^1^. Unfortunately, notwithstanding major advances in glucose-lowering therapies, the prevalence of DM and the accompanying global burden have been increasing over the past few decades, and this rise is expected to continue in the coming years^2^.

Sodium-glucose co-transporter 2 (SGLT2) inhibitors promote a decrease in renal glucose reabsorption in the proximal tubules of the kidneys, thereby increasing urinary glucose excretion^3^. Multiple clinical trials of SGLT2 inhibitors have consistently demonstrated that they can reduce cardiovascular events and improve renal outcomes in type 2 DM (T2DM) patients^4,5^. On the basis of favorable results from previous trials, current international guidelines for the management of T2DM recommend the use of SGLT2 inhibitors in patients with atherosclerotic cardiovascular disease, heart failure, and renal disease^6^. Although the role of SGLT2 inhibitors in organ-protective effects in T2DM patients is being studied currently, the underlying mechanism remains elusive.

Oxidative stress induces dysregulated mitochondrial biogenesis, increases the mitochondrial DNA (mtDNA) copy numbers, and eventually has been implicated in T2DM and related complications^7^. Damaged mtDNA activates the Toll-like receptor 9 pathway and ultimately promotes chronic inflammation^7^. Consequently, urinary and circulating mtDNA are emerging as novel, easily accessible, and noninvasive biomarkers for detecting mitochondrial dysfunction in many clinical scenarios^8–14^. Nicotinamide adenine dinucleotide dehydrogenase subunit-1 (mtND-1) and cytochrome-c oxidase 3 (mtCOX-3) genes encode subunits of the mitochondrial respiratory chain, complex I and complex IV, respectively^15^. Furthermore, these genes are located at sites opposite each other on the circular mtDNA^15^. They therefore represent mtDNA functionally and anatomically in particular^10^.

The association between chronic inflammation and T2DM is now well established, and interleukin-1β (IL-1β), a pro-inflammatory cytokine, acts as a driving force in the pathogenesis of T2DM and related complications^16–18^. Overproduction of mitochondrial reactive oxygen species plays a key role in chronic inflammation through Nod-like receptor protein-3 (NLRP3) inflammasome activation^19^. NLRP3 inflammasome recognizes the damage-associated molecular patterns and leads to caspase-1 activation, and subsequently, to IL-1β maturation and secretion^20^. NLRP3 and IL-1β play a pivotal role in mitochondrial membrane permeability transition and release of mtDNA into the cytosol; the released mtDNA binds to and activates NLRP3, indicating that they have an impact on each other under oxidative stress conditions, and thus, contribute to the excessive secretion of IL-1β^21–23^.

Evidence from animal models helps to elucidate the mechanisms underpinning the beneficial effects of SGLT2 inhibitors, through amelioration of mitochondrial damage and inflammation^24–27^. Improving mitochondrial impairment and ameliorating inflammation could potentially contribute to organ protection^25,28^. SGLT2 inhibitors restore mitochondrial dynamics and mitophagy and suppress mitochondrial oxidative stress in the renal tubules in a mouse model of high-fat diet-induced obesity^24^ and the myocardium in mouse diabetes models^25,26^. SGLT2 inhibitor-mediated alleviation of mitochondrial dysfunction promotes recovery from proximal tubular injury and myocardial microvascular damage^24,25^. SGLT2 inhibitors also attenuate mRNA and protein level expression of inflammatory markers including NLRP3, IL-1β, and tumor necrosis factor-α in kidney and myocardium of a diabetic mouse model, and retard the progression of diabetic kidney disease and cardiomyopathy^29,30^. However, to date, the beneficial effects of SGLT2 inhibitors on mitochondrial damage and inflammation have not been completely clarified in clinical trials.

Thus, the aim of the present study is to explore the favorable effects of empagliflozin, a SGLT2 inhibitor, against mitochondrial dysfunction and chronic inflammation in T2DM patients.

## Methods

### Study populations

We prospectively recruited healthy volunteers (*n* = 22), and SGLT2 inhibitor-naïve T2DM patients (*n* = 21) at the Soonchunhyang University Hospital (Seoul, South Korea). Healthy volunteers (≥ 18 years old) were sorted into groups by medical history, physical examination, and laboratory tests. In the SGLT2 inhibitor-naïve T2DM group, the eligible patients were adults (≥ 18 years old) who were either naïve with respect to glucose-lowering agents (*n* = 2) or had undergone treatment with stable glucose-lowering therapy for at least 12 weeks before enrollment (*n* = 19).

Patients who had type 1 DM, uncontrolled DM requiring immediate intensive therapy, major cardiovascular or cerebrovascular events in the 6 months preceding the study, estimated glomerular filtration rate (eGFR) < 45 mL/min/1.73 m^2^, and those who were pregnant or had undergone a kidney transplant were excluded.

### Study design

Eligible patients received 10 mg or 25 mg of empagliflozin once daily (10 mg, *n* = 17 or 25 mg, *n* = 4), as illustrated in Supplementary Fig. S1. The dosage of empagliflozin necessary to achieve glycemic control was decided at the discretion of the treating physician. The use of other glucose-lowering agents and control of cardiovascular risk factors were also at the discretion of the treating physician, in accordance with the Korean guidelines.

The study was carried out in accordance with the ethical principles in the Declaration of Helsinki. The protocol was approved by the Institutional Review Board (IRB file number: 2016-07-032) of the Soonchunhyang University Hospital. All the participants provided written informed consent for their participation in the study.

### Clinical data collection and laboratory measurements

We collected demographic information and laboratory measurements of the participants at the start of the study. The eGFR was calculated using the chronic kidney disease epidemiology collaboration (CKD-EPI) formula^31^. Urine samples from all the participants were collected (24 h urine in healthy volunteers and spot urine in T2DM patients), centrifuged, and the supernatants were collected and frozen at − 80 °C until analysis. The follow-up data of T2DM patients were collected 3 months after treatment with empagliflozin.

### Urine and serum mtDNA copy numbers

In all the participants, the mtND-1 and mtCOX-3 copy numbers in urine and serum were measured using quantitative real-time polymerase chain reaction (RT-qPCR), and re-measured 3 months after treatment with empagliflozin in T2DM patients, as previously described^12^. In brief, DNA was isolated and purified from urine (1.75 mL) and serum (200 µL) using DNA isolation kits from Norgen Biotek (Thorold, ON, Canada) and Qiagen (Venlo, Limburg, Netherlands), respectively, according to the manufacturer’s instructions. DNA concentrations were measured using a NanoDrop spectrophotometer (Thermo Fisher Scientific, Waltham, MA, USA). RT-qPCR was conducted using ND-1 primers: forward 5′-AGTCACCCTAGCCATCATTCTACT-3′ and reverse 5′-GGAGTAATCAGAGGTGTTCTTGTGT-3′, and COX-3 primers: forward 5′-AGGCATCACCCCGCTAAATC-3′ and reverse 5′-GGTGAGCTCAGGTGATTGATACTC-3′ (Life Technologies, Carlsbad, CA, USA) with 20 ng of template DNA/sample. The PCR was carried out using the following conditions: 95 °C for 10 min, 40 cycles of 95 °C for 15 s, and 60 °C for 60 s.

To identify mitochondria-specific cellular damage, mtDNA copy numbers were corrected to those of the nuclear control gene, RNase P (Life Technologies), using human genomic DNA for plotting the standard curve. Copy numbers were determined using Copy Caller software (version 2.0, Life Technologies). mtDNA copy numbers were expressed as mtDNA/nuclear DNA ratios^9,12^.

### Urinary IL-1β analysis

Urinary IL-1β levels were measured by enzyme-linked immunosorbent assay (Thermo Fisher Scientific) in all the participants at baseline and 3 months after empagliflozin treatment in patients with T2DM according to the manufacturer’s instructions. Urinary IL-1β levels were corrected to urinary creatinine.

### Statistical analyses

Non-parametric tests were employed owing to the small sample size used in this study. Continuous data were expressed as the median (interquartile range) and compared using the Mann–Whitney *U* test or Wilcoxon signed-rank test. Categorical data were expressed as proportions and compared using the chi-squared test. Correlations were assessed using the Spearman’s rank correlation coefficient. To adjust for possible confounding variables and evaluate the form of association, generalized estimating equations were used with different adjustment parameters: model 1 adjusted for age and sex; model 2 additionally adjusted for body mass index (BMI) and systolic and diastolic blood pressure; while model 3 included further adjustment for glycated hemoglobin (HbA1c), eGFR, and urinary protein and albumin. A *P*-value of less than 0.05 was considered statistically significant. All statistical analyses were performed using the commercially available SPSS 25.0 software package (SPSS, Chicago, IL, USA, www.ibm.com/analytics/spss-statistics-software). GraphPad Prism was used for plotting (version 5.03, GraphPad Software, San Diego, CA, USA, www.graphpad.com).

## Results

### Characteristics of the participants

The baseline demographic characteristics and laboratory measurements of the participants are presented in Table 1. SGLT2 inhibitor-naïve T2DM patients were, on average, older than healthy volunteers and had higher BMI, systolic blood pressure, fasting blood glucose, and urinary protein and albumin, but lower eGFR. The median duration of diabetes was 6.0 (interquartile range 1.5–16.0) years. At baseline, 10 (47.6%) had a history of hypertension, and 9 (42.9%) were treated with a stable dose of angiotensin II receptor blocker in the T2DM group.

**Table 1:** Baseline characteristics of participants.

Variables	Healthy volunteers (*n* = 22)	Diabetic patients (*n* = 21)	*P* value
Gender (male *n*, %)	8 (36.4%)	13 (61.9%)	0.094
Age (years)	37.50 (27.50–42.25)	51.00 (44.00–59.00)	< 0.001
BMI (kg/m^2^)	22.10 (20.78–23.17)	29.14 (26.41–31.41)	< 0.001
Systolic BP (mmHg)	110.00 (100.00–110.00)	130.00 (120.00–135.50)	< 0.001
Diastolic BP (mmHg)	80.00 (70.00–80.00)	80.00 (70.00–81.50)	0.145
BUN (mg/dL)	11.20 (8.88–12.90)	12.70 (10.10–15.90)	0.117
Serum creatinine (mg/dL)	0.72 (0.67–0.99)	0.81 (0.61–0.95)	0.817
eGFR^a^ (mL/min/1.73 m^2^)	109.87 (99.31–116.24)	104.10 (92.33–109.58)	0.024
Fasting glucose (mg/dL)	95.50 (90.00–101.25)	164.00 (144.00–188.00)	< 0.001
UPCR or 24 h urine protein^b^	58.65 (44.53–77.55)	88.70 (49.00–197.10)	0.016
UACR or 24 h urine albumin^b^	2.00 (1.55–3.08)	17.80 (4.85–56.55)	< 0.001

Values are expressed as *n* (%) for gender and median (interquartile range) for continuous data.

*BMI* body mass index, *BP* blood pressure, *BUN* blood urea nitrogen, *eGFR* estimated glomerular filtration rate, *UACR* urinary albumin to creatinine ratio, *UPCR* urinary protein to creatinine ratio.

^a^The eGFR was calculated using the chronic kidney disease epidemiology collaboration (CKD-EPI) formula.

^b^The 24 h urine protein and albumin (mg/day) were tested in healthy volunteers, and spot urine protein and albumin to creatinine ratio (mg/g) were evaluated in diabetes patients.

### Profiles of glucose-lowering medication

Table 2 shows the medication used to manage diabetes before and after empagliflozin treatment. Because a combination of dipeptidyl peptidase-4 (DPP-4) inhibitor and SGLT2 inhibitor was not covered by insurance in Korea during the study period, DPP-4 inhibitors were the drugs most frequently replaced by empagliflozin, followed by sulfonylurea. Most patients continued metformin treatment during the study period.

**Table 2:** Change in demographic and clinical parameters after SGLT2 inhibitor treatment.

Variables	Before (*n* = 21)	After (*n* = 21)	*P* value
**Medication (*n*, %)**			
Metformin	18 (85.7%)	19 (90.5%)	1.000
DPP-4 inhibitor	10 (47.6%)	0 (0.0%)	< 0.001
Sulfonylurea	10 (47.6%)	7 (33.3%)	0.346
Insulin	4 (19.0%)	3 (14.3%)	1.000
BMI (kg/m^2^)	29.14 (26.41–31.41)	28.07 (24.85–30.67)	0.001
Systolic BP (mmHg)	130.00 (120.00–135.50)	130.00 (120.00–139.00)	0.531
Diastolic BP (mmHg)	80.00 (70.00–81.50)	80.00 (71.00–90.00)	0.420
BUN (mg/dL)	12.70 (10.10–15.90)	13.90 (11.50–17.15)	0.024
Serum creatinine (mg/dL)	0.81 (0.61–0.95)	0.79 (0.62–0.96)	0.468
eGFR^a^ (mL/min/1.73 m^2^)	104.10 (92.33–109.58)	101.24 (92.61–108.36)	0.398
Fasting glucose (mg/dL)	164.00 (144.00–188.00)	144.00 (134.50–170.00)	0.006
HbA1c (%)	8.00 (7.10–8.70)	7.50 (6.70–8.20)	0.040
HbA1c (mmol/mol)	64.00 (54.00–72.00)	58.00 (50.00–66.00)	0.042
UPCR	88.70 (49.00–197.10)	117.20 (69.40–235.75)	0.520
UACR	17.80 (4.85–56.55)	39.00 (4.80–117.00)	0.394

Values are expressed as *n* (%) for categorical data and median (interquartile range) for continuous data.

*BMI* body mass index, *BP* blood pressure, *BUN* blood urea nitrogen, *DPP-4* dipeptidyl peptidase-4, *eGFR* estimated glomerular filtration rate, *HbA1c* glycated hemoglobin, *SGLT2* sodium-glucose co-transporter 2, *UACR* urinary albumin to creatinine ratio, *UPCR* urinary protein to creatinine ratio.

^a^The eGFR was calculated using the chronic kidney disease epidemiology collaboration (CKD-EPI) formula.

### Cell-free mtDNA copy numbers in T2DM patients

**Figure 1:**
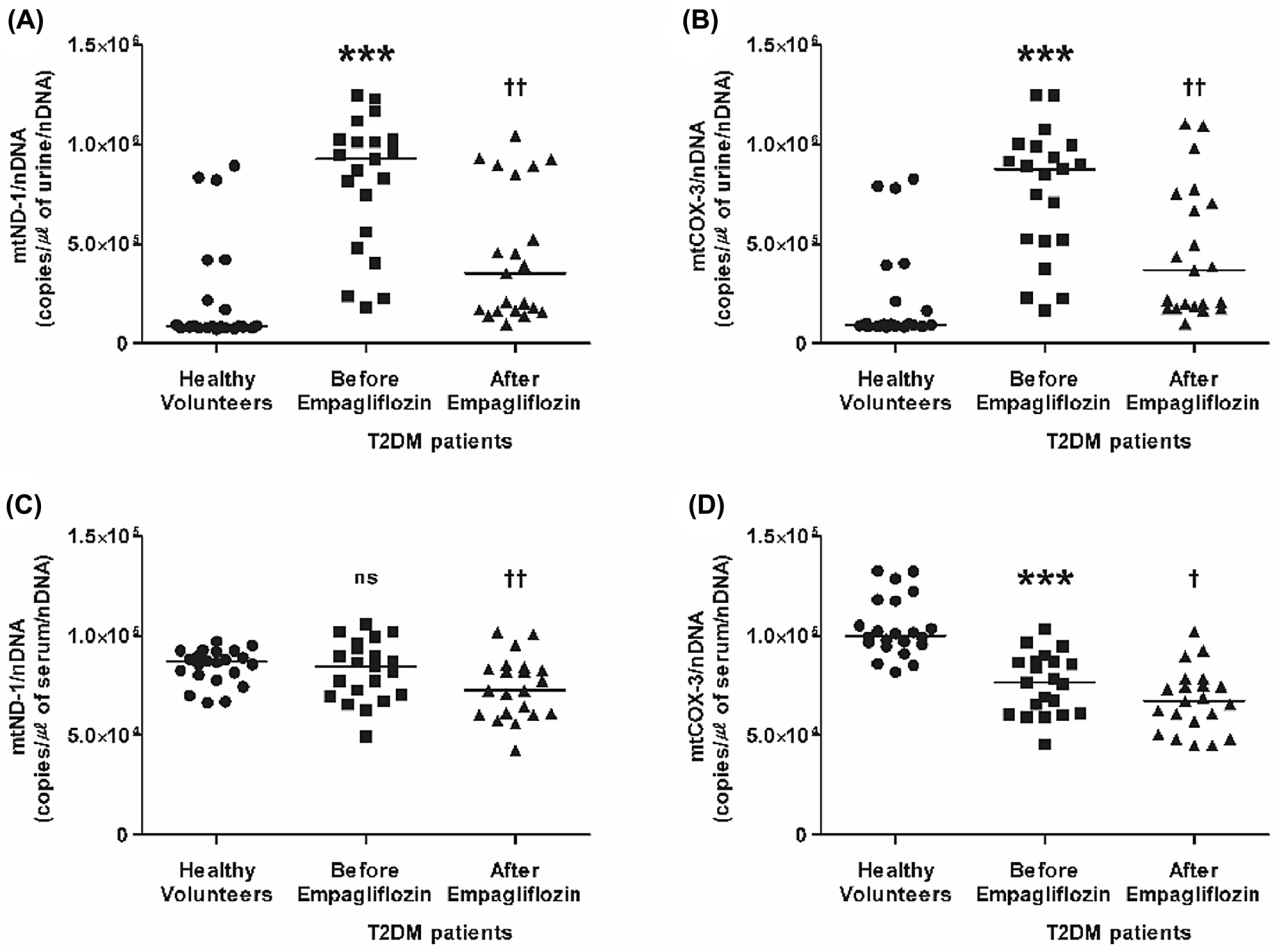
Urinary and circulating mtDNA copy numbers in healthy volunteers (*n* = 22) and T2DM patients (*n* = 21) before and after empagliflozin treatment. (**A**) Urinary mtND-1, (**B**) urinary mtCOX-3, (**C**) circulating mtND-1, and (**D**) circulating mtCOX-3. Horizontal line represents median. ***P < 0.001 vs. healthy volunteers, ^†^*P* < 0.05 vs. before empagliflozin treatment, ^††^*P* < 0.01 vs. before empagliflozin treatment. Statistical analysis was performed using SPSS (version 25.0, SPSS, Chicago, IL, USA, www.ibm.com/analytics/spss-statistics-software) and figure was plotted using GraphPad Prism (version 5.03, GraphPad Software, San Diego, CA, USA, www.graphpad.com). *mtCOX-3* cytochrome-c oxidase 3, *mtDNA* mitochondrial DNA, *mtND-1* nicotinamide adenine dinucleotide dehydrogenase subunit-1, *nDNA* nuclear DNA, *ns* not significant, *T2DM* type 2 diabetes mellitus.

The urinary mtND-1 and mtCOX-3 copy numbers were remarkably higher in T2DM patients than in healthy volunteers (Fig. 1A,B). However, while serum mtND-1 copy numbers in T2DM patients did not differ from healthy volunteers (Fig. 1C, P = 0.680), the serum mtCOX-3 copy numbers were lower than those in healthy volunteers (Fig. 1D).

**Figure 2:**
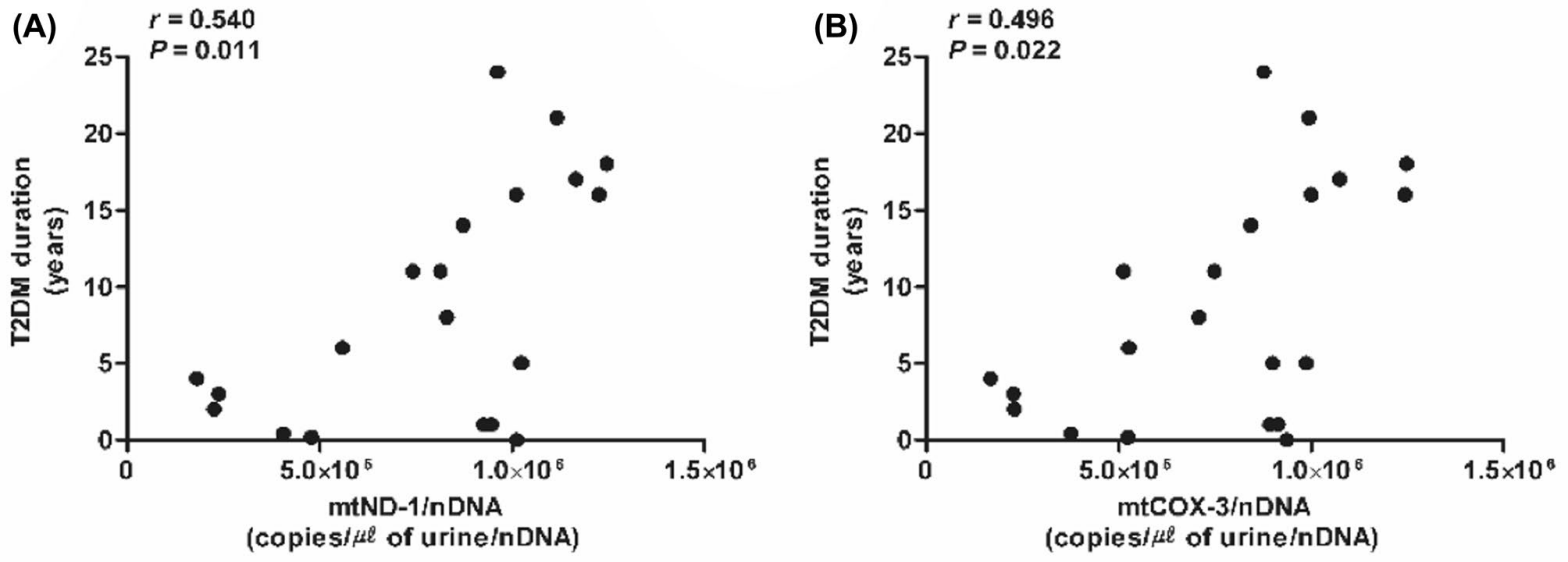
Correlation between urinary mtDNA copy numbers and T2DM duration (*n* = 21). (**A**) urinary mtND-1, and (**B**) urinary mtCOX-3. Statistical analysis was performed using SPSS (version 25.0, SPSS, Chicago, IL, USA, www.ibm.com/analytics/spss-statistics-software) and figure was plotted using GraphPad Prism (version 5.03, GraphPad Software, San Diego, CA, USA, www.graphpad.com). *mtCOX-3* cytochrome-c oxidase 3, *mtDNA* mitochondrial DNA, *mtND-1* nicotinamide adenine dinucleotide dehydrogenase subunit-1, *nDNA* nuclear DNA, *T2DM* type 2 diabetes mellitus.

Notably, urinary mtDNA copy numbers were associated with T2DM duration (Fig. 2A,B) and this association remained significant after an adjustment for possible confounders (Supplementary Table S1). When divided into two subgroups according to T2DM duration at a cut-off of 5 years, even patients with a short duration of T2DM showed high urinary mtDNA copy numbers (Supplementary Fig. S2). Serum mtCOX-3 copy numbers decreased in the early years of T2DM, while those of serum mtND-1 did not (Supplementary Fig. S2).

In addition, urinary mtDNA copy numbers showed a negative correlation with blood pressure (Supplementary Fig. S3). However, they did not correlate with age, BMI, HbA1c, eGFR, or urinary protein and albumin (data not shown). Moreover, there was no correlation between urinary and serum mtDNA copy numbers (*r* = 0.149, *P* = 0.518 for mtND-1; *r* = 0.091, *P* = 0.695 for mtCOX-3).

### Urinary IL-1β in T2DM patients

**Figure 3:**
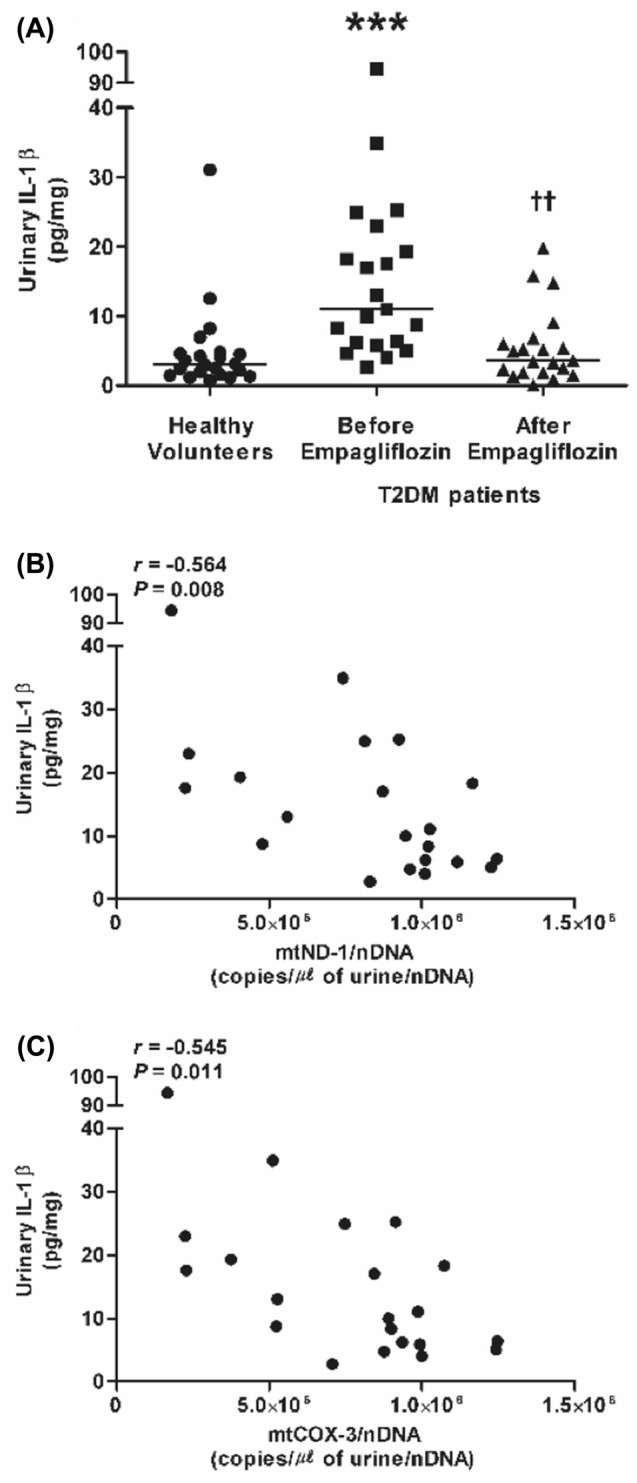
Urinary IL-1β in healthy volunteers (*n* = 22) and T2DM patients (*n* = 21) before and after empagliflozin treatment. (**A**) urinary IL-1β, (**B**) correlation with urinary mtND-1, and (**C**) urinary mtCOX-3. Horizontal line represents median. ***P < 0.001 vs. healthy volunteers, ^††^*P* < 0.01 vs. before empagliflozin treatment. Statistical analysis was performed using SPSS (version 25.0, SPSS, Chicago, IL, USA, www.ibm.com/analytics/spss-statistics-software) and figure was plotted using GraphPad Prism (version 5.03, GraphPad Software, San Diego, CA, USA, www.graphpad.com). *IL-1β* interleukin-1β, *mtCOX-3* cytochrome-c oxidase 3, *mtND-1* nicotinamide adenine dinucleotide dehydrogenase subunit-1, *nDNA* nuclear DNA, *T2DM* type 2 diabetes mellitus.

Urinary IL-1β levels were elevated in the T2DM group (Fig. 3A), and correlated inversely with urinary mtND-1 and mtCOX-3 copy numbers (Fig. 3B,C). As with urinary mtDNA copy numbers, urinary IL-1β also did not show a significant correlation with age, BMI, HbA1c, eGFR, or urinary protein and albumin (data not shown).

### Changes in mtDNA copy numbers and urinary IL-1β in empagliflozin-treated T2DM patients

After 3 months of empagliflozin treatment, the BMI, fasting blood glucose, and HbA1c values significantly decreased; however, the systolic and diastolic blood pressure, serum creatinine, eGFR, and urinary protein and albumin were comparable (Table 2).

After empagliflozin treatment, the copy numbers of urinary mtND-1 and mtCOX-3 (Fig. 1A,B), as well as those of serum mtND-1 and mtCOX-3 (Fig. 1C,D), were significantly decreased. Moreover, urinary IL-1β was significantly reduced after empagliflozin treatment (Fig. 3A).

### Association of clinical parameters with changes in mtDNA copy numbers or urinary IL-1β

A decrease in urinary mtDNA copy numbers was not significantly associated with changes in BMI, blood pressure, or glycemic control (Supplementary Fig. S4). The change in urinary IL-1β correlated with change in systolic blood pressure, but did not correlate with changes in BMI, diastolic blood pressure, or HbA1c (Supplementary Fig. S5). The degree of reduction in urinary and serum mtDNA copy numbers and urinary IL-1β did not differ with the dose of empagliflozin (data not shown). Change in urinary IL-1β was negatively associated with change in urinary mtCOX-3 copy number (*r* = − 0.524, *P* = 0.015), but not with change in the copy numbers of urinary mtND-1 (*r* = − 0.392, *P* = 0.079), and serum mtDNA (*r* = − 0.185, *P* = 0.421 for mtND-1, *r* = − 0.057, *P* = 0.805 for mtCOX-3).

### Association of markers of kidney disease with changes in mtDNA copy numbers or urinary IL-1β

To examine the association among the changes in mitochondrial damage-associated molecular patterns, pro-inflammatory cytokine, and traditional markers of kidney damage, we evaluated the correlation along with a change in eGFR or albuminuria. Only a change in serum mtDNA copy numbers was correlated with change in eGFR (Supplementary Fig. S6), whereas changes in urinary mtDNA copy numbers and IL-1β did not correlate with change in eGFR (*r* = − 0.184, *P* = 0.423 for mtND-1; *r* = 0.019, *P* = 0.935 for mtCOX-3; *r* = 0.095, *P* = 0.681 for IL-1β). A change in albuminuria was not associated with changes in either urinary mtDNA copy numbers (*r* = 0.164, *P* = 0.477 for mtND-1; *r* = 0.077, *P* = 0.739 for mtCOX-3) or those in serum (*r* = − 0.110, *P* = 0.635 for mtND-1; *r* = − 0.035, *P* = 0.879 for mtCOX-3), or urinary IL-1β (*r* = − 0.066, *P* = 0.778).

## Discussion

In this study, we demonstrated that empagliflozin showed a beneficial effect towards decreasing urinary and circulating cell-free mtDNA copy numbers and urinary IL-1β in patients with T2DM. Urinary mtDNA copy numbers and IL-1β were elevated in T2DM patients and they correlated with each other. Although BMI and glycemic control were improved after treatment with empagliflozin, the weight loss and glycemic control were not associated with a reduction in urinary mtDNA copy numbers and IL-1β, implying that empagliflozin contributes to the improvement in mitochondrial damage and inflammation through a mechanism separate from that of weight loss or glycemic control.

Increasing evidence suggests that SGLT2 inhibitors have beneficial effects in the cardiovascular and renal outcomes by improving mitochondrial homeostasis and suppressing oxidative stress^3,24–26^. Notably, SGLT2 inhibitors alleviated inflammation that was partly mediated by attenuation of oxidative stress, thereby inhibiting the progression of diabetic complications^28,32^. Urinary mtDNA copy numbers are extensively used as biomarkers of renal mitochondrial integrity during kidney injury^8–13^. Moreover, it has been reported that urinary mtDNA copy numbers might indicate kidney resilience^8^. Furthermore, IL-1β has gained immense attention as an important risk factor of T2DM^18^. Our previous study identified that a decrease in urinary mtDNA copy numbers correlates with urinary albumin decrement after bariatric surgery in obese patients^12^. Markers of renal injury in patients with T2DM independently predict increased cardiovascular morbidity and mortality^1^. The decrease in elevated urinary mtDNA copy numbers and IL-1β conferred by empagliflozin supports the evidence that amelioration of mitochondrial dysfunction and inflammation could have a beneficial impact on the improved cardiorenal outcomes already revealed in landmark clinical trials with SGLT2 inhibitors^4,5^.

The decrease in urinary IL-1β after empagliflozin treatment is in accordance with the results of the previous randomized, controlled study, which reported that empagliflozin inhibited the release of IL-1β from macrophage in T2DM patients with high cardiovascular risk^33^. In a recent study with T2DM patients not under the influence of renin-angiotensin system inhibition, cell-free circulating mtDNA levels and plasma IL-1β were elevated and showed a weak correlation^34^. Interestingly, urinary cell-free mtDNA copy numbers were found to be negatively correlated with urinary IL-1β in the present study. These controversial results might be attributable to differences in the characteristics of the study population. Unlike the above-mentioned study, 42.9% of patients were being treated with angiotensin II receptor blocker in our study. T2DM duration and the anti-diabetic medications taken by participants at the initiation of this study may have influenced the outcome. Additionally, there were differences in the experimental protocol (type of analyzed samples, mtDNA level measurement method). However, urinary cell-free mtDNA copy numbers and IL-1β were elevated in T2DM patients, indicating that T2DM patients are exposed to mitochondrial damage and inflammation.

In the present study, T2DM patients showed small but statistically significant weight loss (median = 0.20 kg, *P* = 0.001) and HbA1c reduction upon 3 months of empagliflozin treatment. Despite the small weight loss, empagliflozin considerably reduced cell-free mtDNA copy numbers in T2DM patients compared to those in non-diabetic obese patients who had undergone bariatric surgery^12^. Moreover, a change in urinary mtDNA copy numbers and IL-1β did not correlate with weight loss or glycemic control in our results. Consistent with our results, SGLT2 inhibitors attenuated oxidative stress and inflammation, independent of glycemic control or weight loss in experimental and clinical studies^24,33,35^. SGLT2 inhibitors induce a fuel switch from glucose to free fatty acid and improve metabolic flexibility and insulin sensitivity^36,37^. This leads to reinstatement of mitochondrial biogenesis, mammalian target of rapamycin inhibition, and activation of autophagy and lysosomal degradation, resulting in the removal of damaged mitochondria^37^. Moreover, the fuel switch promotes ketone production, which is known to block NLRP3 inflammasome^36,38^. Ketones specifically suppress NLRP3 inflammasome activation by inhibiting K^+^ efflux and inflammasome assembly, and subsequent IL-1β secretion^38^. SGLT2 inhibitor attenuated NLRP3 inflammasome activation in a mouse model of T2DM independent of its anti-diabetic effects^29^. An inhibitory effect of ketone on NLRP3 inflammasome activation and IL-1β secretion has been seen in human macrophages treated with SGLT2 inhibitor independent of glucose-lowering and weight loss^33^. Although glycemic control and weight loss have been considered important for the prevention of diabetic complications, SGLT2 inhibitors protect against organ damage not only through these favorable effects, but also through amelioration of mitochondrial perturbation and inflammation.

The duration of diabetes is related to the risk of vascular complications in T2DM patients^39^. Urinary mtDNA copy numbers were correlated with T2DM duration in this study, which is in accordance with the results of a recent study reported by Cao et al.^40^ These copy numbers were elevated even in T2DM patients who had diabetes for less than 5 years in our study. These data highlight that elevated urinary mtDNA copy numbers could be used as sensitive markers of T2DM and may reflect a high risk of vascular complications.

We found a decrease in the serum mtCOX-3 copy numbers in T2DM patients. This finding is in accordance with the result of previous studies involving obese and hypertensive patients^10,12^. Although circulating cell-free mtDNA is expected to originate from damaged cells^41^, the mechanism by which it is released into circulation is not yet clear, and the clinical implications in T2DM patients are controversial^34,40,42,43^. Our findings are consistent with previous data showing that circulating mtDNA copy numbers were decreased and those of urinary mtDNA were increased in T2DM patients^40^. The authors in the aforementioned study inferred that circulating mtDNA undergoes filtration and passes into the urine^40^. In another study, the authors suggested that a small fraction of circulating DNA passes through the kidneys^44^, and therefore, the proportion of mtDNA to be filtered is considered low. In our study, serum mtDNA copy numbers did not correlate with urinary mtDNA copy numbers, and this result is in keeping with our previous study on obesity^12^. Therefore, hyperfiltration does not fully explain the observed decrease in circulating mtDNA copy numbers. The levels of both serum mtND-1 and mtCOX-3 further decreased after empagliflozin treatment. Investigations into the effect of SGLT2 inhibitors on the mitochondrial respiratory chain are scarce but it has been reported that the beneficial effect of canagliflozin is mediated by mitochondrial respiratory chain complex I inhibition^45^. Further studies are warranted to establish why serum mtDNA copy numbers are lower in T2DM patients and to demonstrate different reactions to empagliflozin treatment.

Although the results were encouraging, our study has certain limitations. The sample size of healthy control participants and T2DM patients was small and the study was carried out for only a short duration. Urine samples were collected differently from healthy controls and diabetic patients in the present study. Previous studies have consistently reported that urinary mtDNA is higher in the diseased group than in the healthy controls despite differences between patient groups and healthy controls in how urine was collected^9,10^. Moreover, our study lacks a group that can be compared with the T2DM group treated with glucose-lowering agents other than SGLT inhibitors. Eventually, we could not perform an experiment to directly identify the restoration mechanism of mitochondrial damage and inflammation in T2DM patients treated with empagliflozin. Further research is warranted to understand the mechanisms underpinning the beneficial effects of SGLT2 inhibitors in T2DM.

In conclusion, empagliflozin reduces urinary and circulating cell-free mtDNA copy numbers and urinary IL-1β in patients with T2DM. These results suggest a possible benefit of SGLT2 inhibitors towards amelioration of mitochondrial damage and reversion of inflammation.

## Supplementary Information

Supplementary Information.

## Data Availability

The data underlying this article cannot be shared publicly to protect the privacy of individuals that participated in the study. The data will be shared on reasonable request to the corresponding author.
