# Supplementary material for: Empagliflozin suppresses urinary mitochondrial DNA copy numbers and interleukin-1β in type 2 diabetes patients
Source: Sci Rep. 2022 Nov 9;12:19103. doi: 10.1038/s41598-022-22083-6 (PMC9646895; doi:10.1038/s41598-022-22083-6)
Supplement: Supplementary file 1 — Supplementary Information. [file 41598_2022_22083_MOESM1_ESM.pdf]

Supplementary Information

Empagliflozin Suppresses Urinary Mitochondrial DNA Copy Numbers and Interleukin-1β in Type 2 Diabetes Patients

Haekyung Lee[1], Hyoungnae Kim[1,2], Jin Seok Jeon[1,2], Hyunjin Noh[1,2], Rojin Park[3], Dong Won Byun[4], Hye Jeong Kim[4], Kyoil Suh[4], Hyeong Kyu Park[4,*] and Soon Hyo Kwon[1,2,*]

[1]Division of Nephrology, Department of Internal Medicine, Soonchunhyang University Seoul Hospital, 59 Daesagwan-ro, Yongsan-gu, Seoul, 04401, Republic of Korea

[2]Hyonam Kidney Laboratory, Soonchunhyang University Seoul Hospital, 59 Daesagwan-ro, Yongsan-gu, Seoul, 04401, Republic of Korea

[3]Department of Laboratory Medicine, Soonchunhyang University Seoul Hospital, 59 Daesagwan-ro, Yongsan-gu, Seoul, 04401, Republic of Korea

[4]Division of Endocrinology and Metabolism, Department of Internal Medicine, Soonchunhyang University Seoul Hospital, 59 Daesagwan-ro, Yongsan-gu, Seoul, 04401, Republic of Korea

Table of Contents

1. Supplementary Figure……………………………………………………………2
2. Supplementary Table………………………………………………………………9

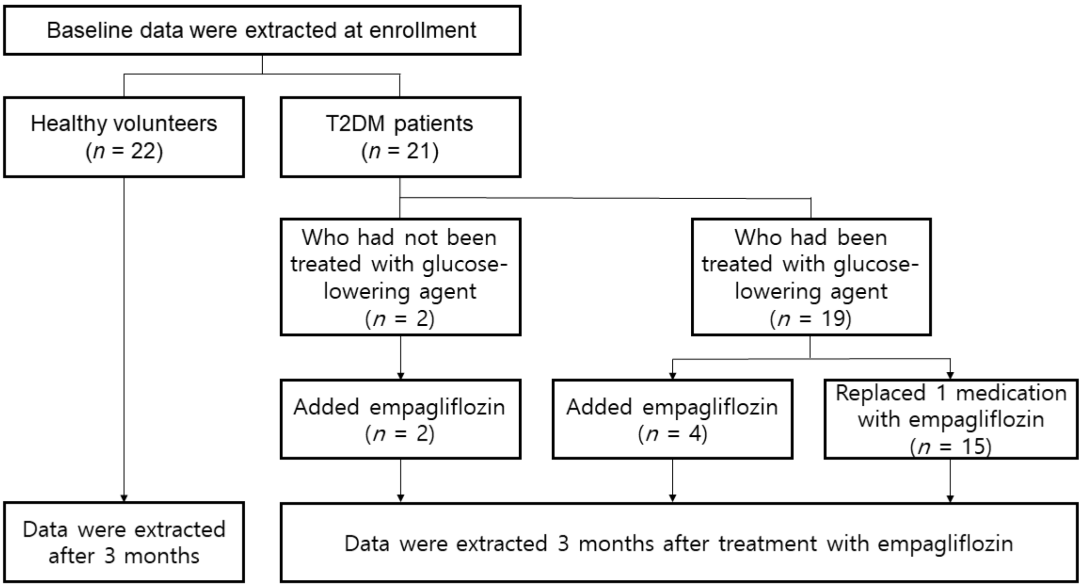

Supplementary Figure S1. Study scheme.

Abbreviations: T2DM, type 2 diabetes mellitus.

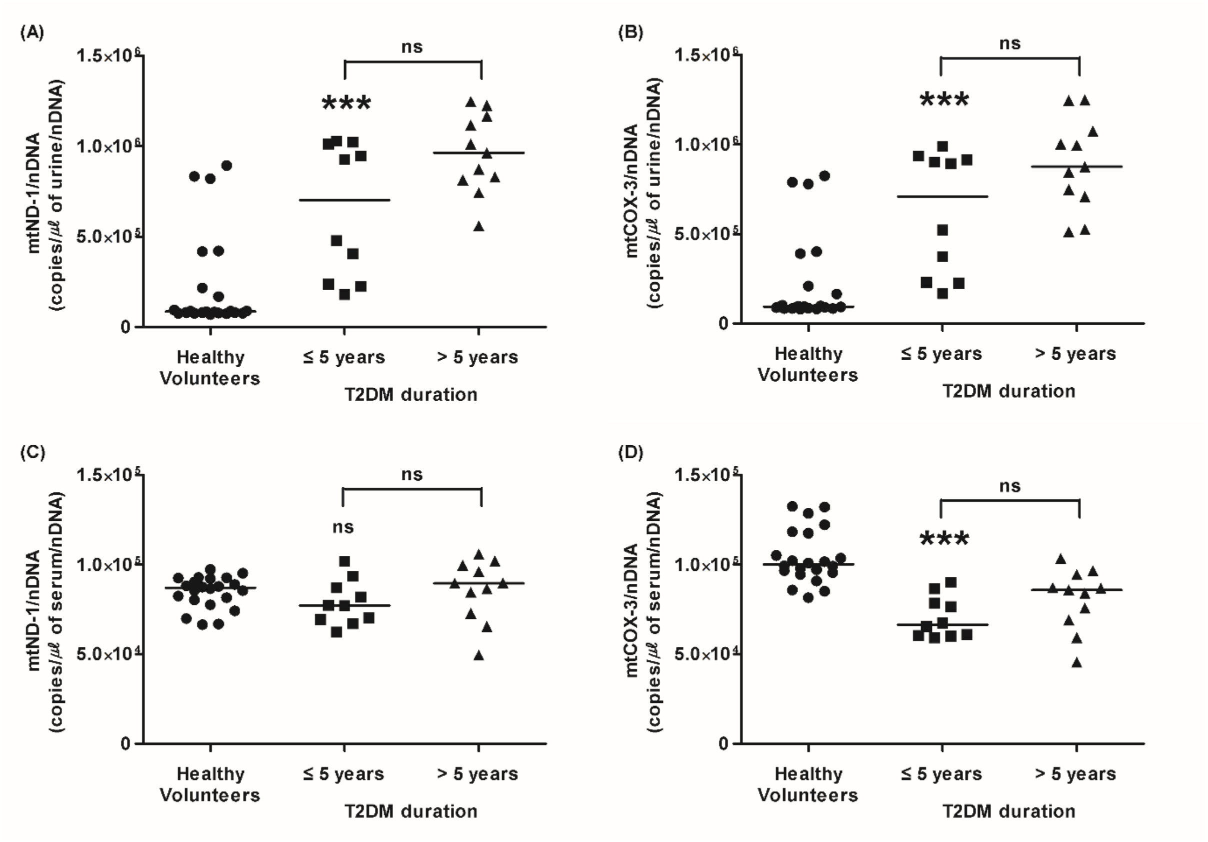

Supplementary Figure S2. Subgroup analysis of mtDNA copy numbers based on T2DM duration.

T2DM patients were divided into two groups based on T2DM duration (≤ 5 years, *n* = 10; > 5 years, *n* = 11). Copy numbers of (A) urinary mtND-1, (B) urinary mtCOX-3, (C) circulating mtND-1, and (D) circulating mtCOX-3. Horizontal line represents median. ****P* < 0.001 vs. healthy volunteers.

Abbreviations: mtCOX-3, cytochrome-c oxidase 3; mtDNA, mitochondrial DNA; mtND-1, nicotinamide adenine dinucleotide dehydrogenase subunit-1; nDNA, nuclear DNA; ns, not significant; T2DM, type 2 diabetes mellitus.

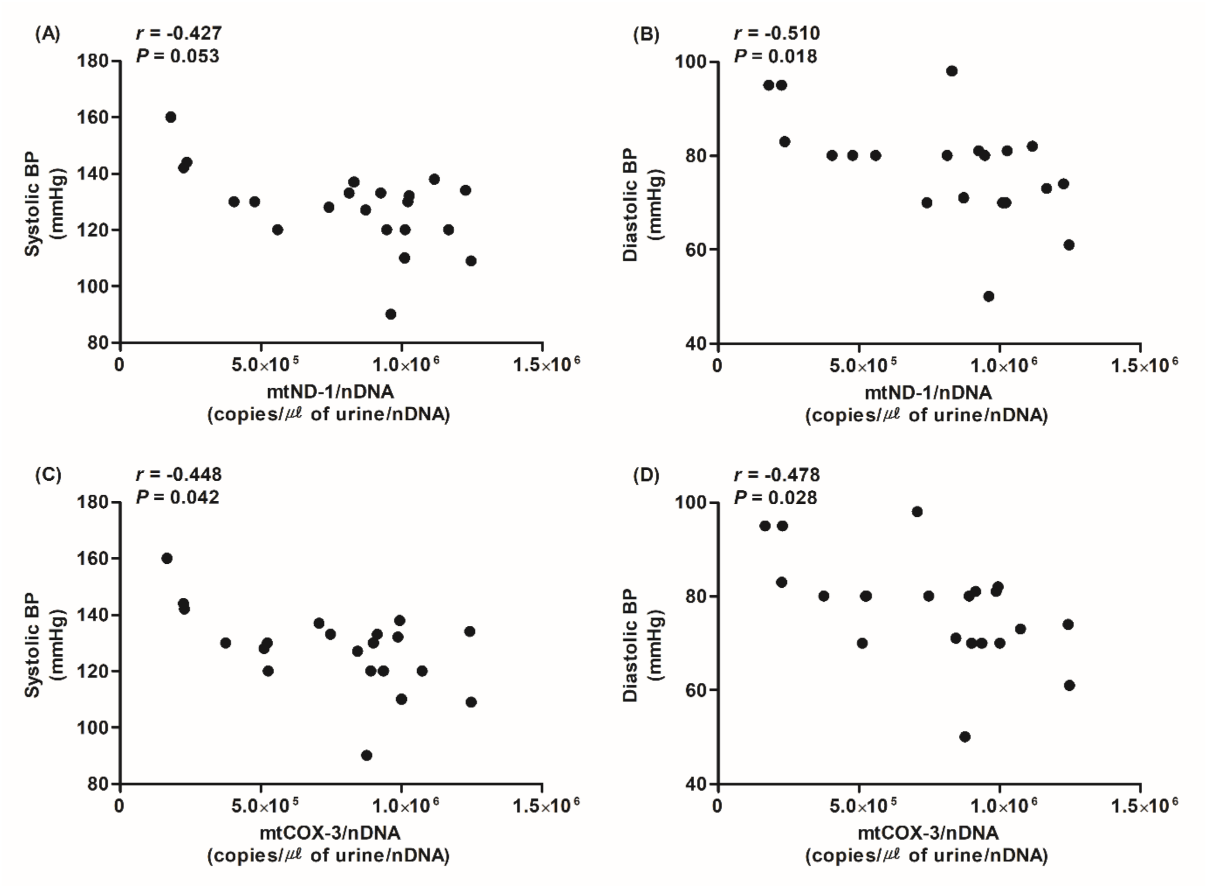

Supplementary Figure S3. Correlation between urinary mtDNA copy numbers and BP.

Urinary mtND-1 copy numbers were inversely correlated with (A) systolic and (B) diastolic BP. In addition, urinary mtCOX-3 also showed an inverse correlation with (C) systolic and (D) diastolic BP.

Abbreviations: BP, blood pressure; mtCOX-3, cytochrome-c oxidase 3; mtDNA, mitochondrial DNA; mtND-1, nicotinamide adenine dinucleotide dehydrogenase subunit-1; nDNA, nuclear DNA.

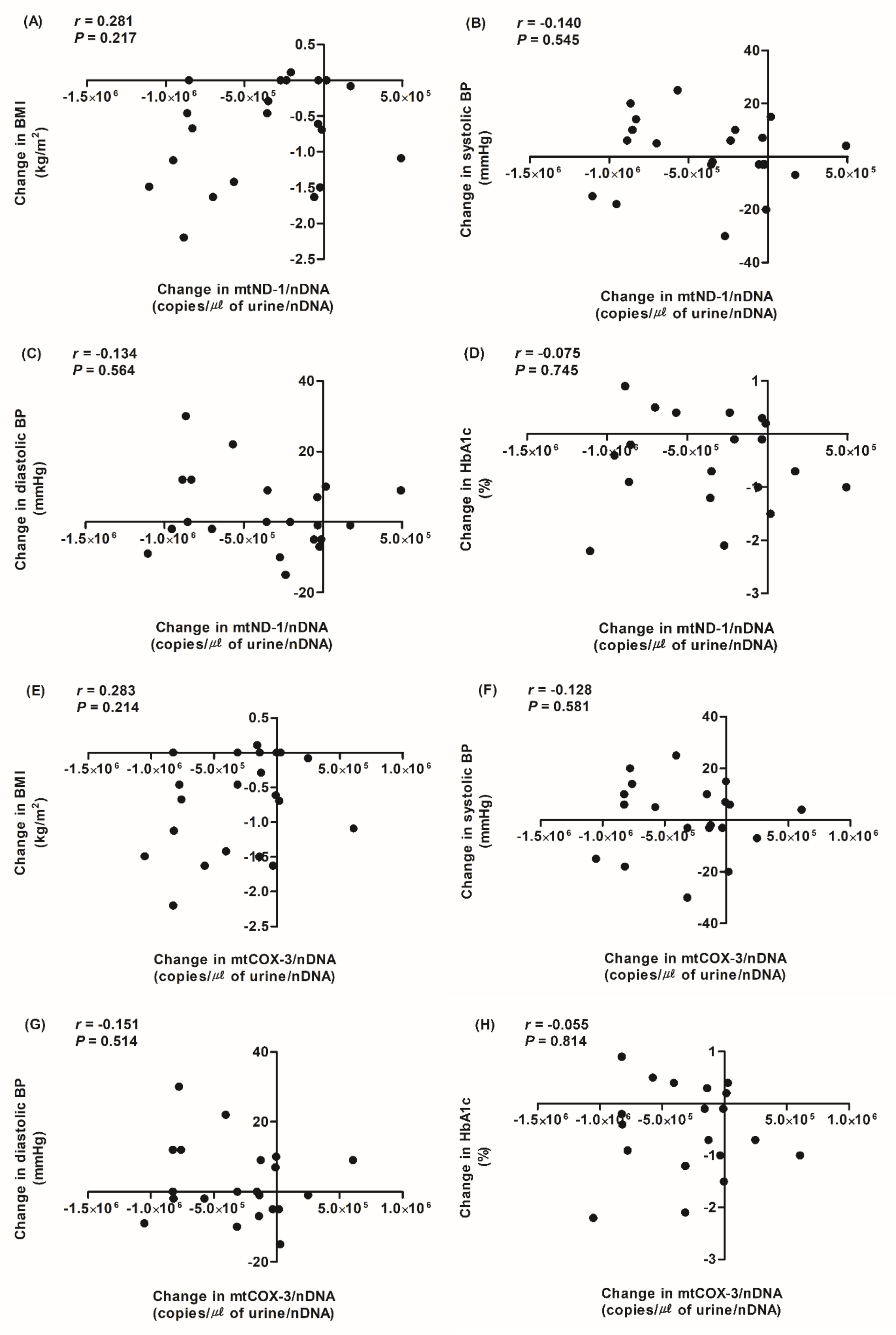

(A)
r = 0.281
P = 0.217
Change in BMI (kg/m2)
Change in mtND-1/nDNA (copies/μl of urine/nDNA)
(B)
r = -0.140
P = 0.545
Change in systolic BP (mmHg)
Change in mtND-1/nDNA (copies/μl of urine/nDNA)
(C)
r = -0.134
P = 0.564
Change in diastolic BP (mmHg)
Change in mtND-1/nDNA (copies/μl of urine/nDNA)
(D)
r = -0.075
P = 0.745
Change in HbA1c (%)
Change in mtND-1/nDNA (copies/μl of urine/nDNA)
(E)
r = 0.283
P = 0.214
Change in BMI (kg/m2)
Change in mtCOX-3/nDNA (copies/μl of urine/nDNA)
(F)
r = -0.128
P = 0.581
Change in systolic BP (mmHg)
Change in mtCOX-3/nDNA (copies/μl of urine/nDNA)
(G)
r = -0.151
P = 0.514
Change in diastolic BP (mmHg)
Change in mtCOX-3/nDNA (copies/μl of urine/nDNA)
(H)
r = -0.055
P = 0.814
Change in HbA1c (%)
Change in mtCOX-3/nDNA (copies/μl of urine/nDNA)

Supplementary Figure S4. Correlation between change in urinary mtDNA copy numbers and alteration in the clinical parameters.

Change in urinary mtND-1 copy numbers showed no correlation with alteration in (A) BMI, (B,C) systolic and diastolic BP, and (D) HbA1c. Change in urinary mtCOX-3 copy numbers also did not correlate with change in (E) BMI, (F,G) systolic and diastolic BP, and (H) HbA1c.

Abbreviations: BMI, body mass index; BP, blood pressure; HbA1c, glycated hemoglobin; mtCOX-3, cytochrome-c oxidase 3; mtDNA, mitochondrial DNA; mtND-1, nicotinamide adenine dinucleotide dehydrogenase subunit-1; nDNA, nuclear DNA.

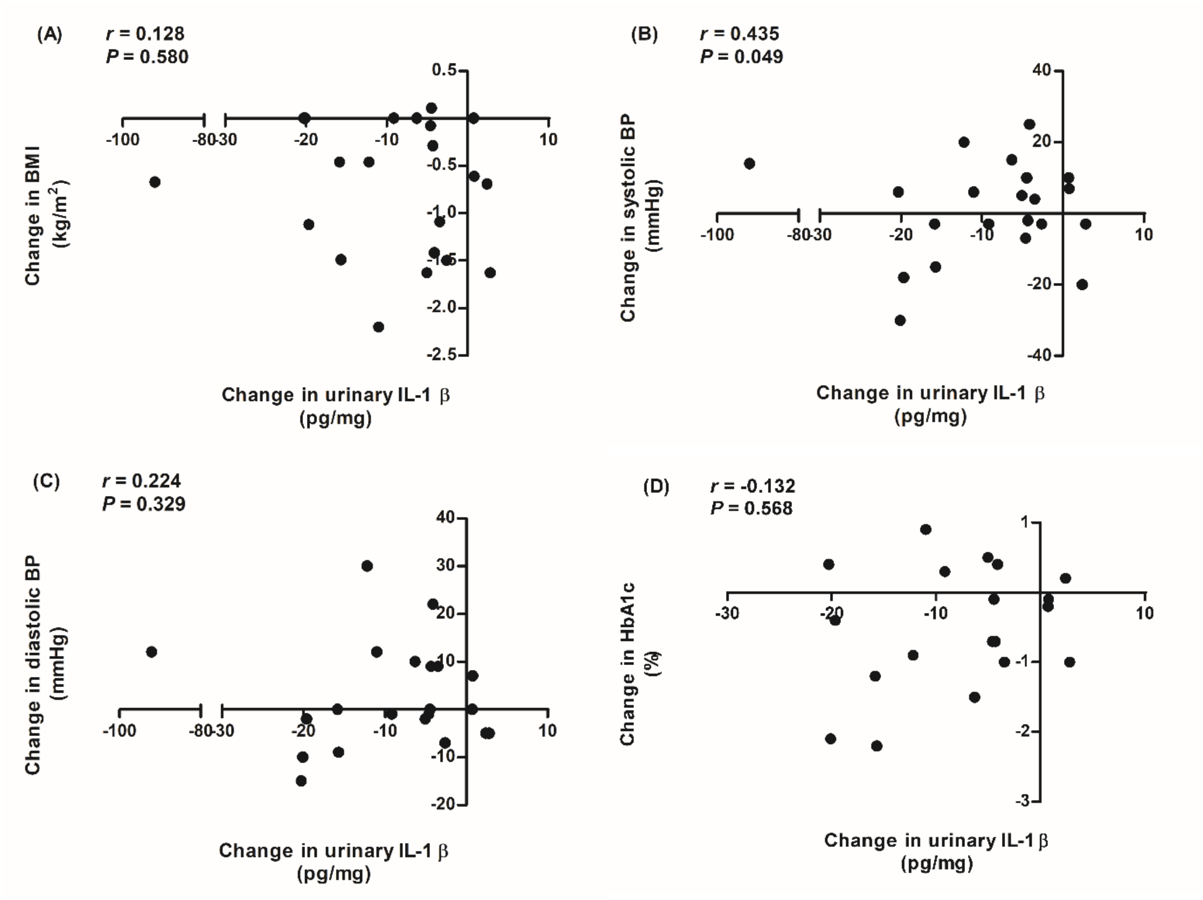

Supplementary Figure S5. Correlation between change in urinary IL-1β and alteration in the clinical parameters.

Change in (A) BMI, (B) systolic BP, (C) diastolic BP, and (D) HbA1c.

Abbreviations: BMI, body mass index; BP, blood pressure; HbA1c, glycated hemoglobin; IL-1β, interleukin-1β.

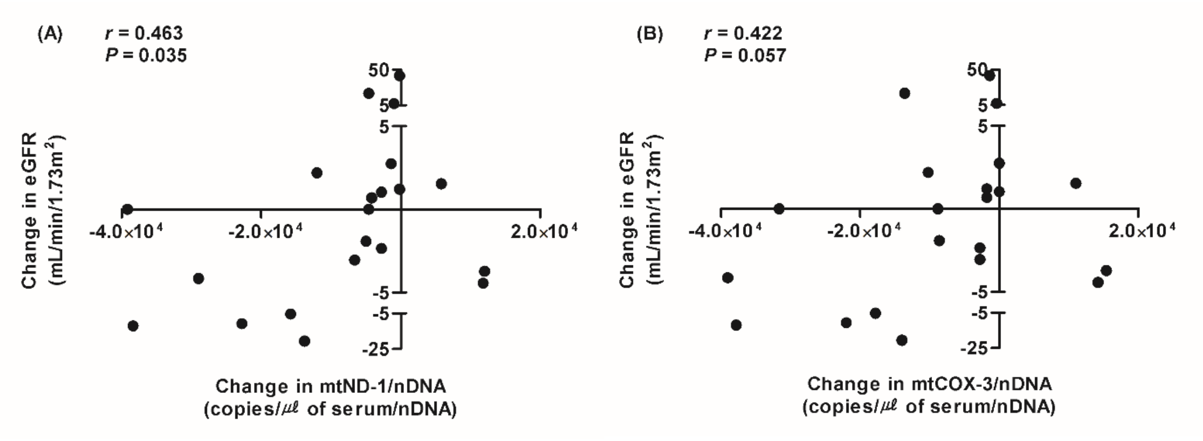

Supplementary Figure S6. Correlation between change in serum mtDNA copy numbers and change in eGFR. Change in circulating (A) mtND-1 and (B) mtCOX-3.

Abbreviations: eGFR, estimated glomerular filtration rate; mtCOX-3, cytochrome-c oxidase 3; mtDNA, mitochondrial DNA; mtND-1, nicotinamide adenine dinucleotide dehydrogenase subunit-1; nDNA, nuclear DNA.

Supplementary Table S1. Association between urinary mtDNA copy numbers and type 2 diabetes duration

Univariable analysis				
		β	SE	*P* value
	mtND-1	25509.44	7411.90	0.001
	mtCOX-3	23450.24	7380.37	0.001
**Multivariable analysis**				
Adjustment model[a]		β	SE	*P* value
Model 1	mtND-1	22844.92	8420.50	0.007
	mtCOX-3	20033.36	8333.65	0.016
Model 2	mtND-1	17987.39	6835.93	0.009
	mtCOX-3	15467.76	5709.58	0.007
Model 3	mtND-1	36592.13	9208.78	<0.001
	mtCOX-3	29370.31	9533.84	0.002

Abbreviations: mtCOX-3, cytochrome-c oxidase 3; mtDNA, mitochondrial DNA; mtND-1, nicotinamide adenine dinucleotide dehydrogenase subunit-1.

[a]Model 1: adjusted for age and sex; Model 2: same as model 1, plus body mass index, systolic blood pressure, and diastolic blood pressure; Model 3: same as model 2, plus glycated hemoglobin, estimated glomerular filtration rate, and urinary protein and albumin.
